# *OsPDIL1-5*: dual role in promoting growth and development while modulating drought stress tolerance in rice (*Oryza sativa* L.)

**DOI:** 10.3389/fpls.2024.1479726

**Published:** 2024-10-15

**Authors:** Jilin Chen, Jin Zhang, Chao Fang, Lijun Ren, Tong Lan, Weiren Wu, Tao Lan

**Affiliations:** ^1^ Key Laboratory of Genetics, Breeding and Multiple Utilization of Crops, Ministry of Education, Fujian Agriculture and Forestry University, Fuzhou, Fujian, China; ^2^ Fujian Provincial Key Laboratory of Crop Breeding by Design, Fujian Agriculture and Forestry University, Fuzhou, Fujian, China; ^3^ Key Laboratory of Biological Breeding for Fujian and Taiwan Crops, Ministry of Agriculture and Rural Affairs, Fujian Agriculture and Forestry University, Fuzhou, Fujian, China

**Keywords:** rice, growth and development, drought tolerance, *GDDT*, *OsPDIL1-5*

## Abstract

Drought tolerance and plant growth are critical factors affecting rice yield, and identifying genes that can enhance these traits is essential for improving crop resilience and productivity. Using a *growth-depressed and drought-tolerant* (*gddt*) mutant of the *indica* rice variety Huanghuazhan (HHZ) generated by radiation mutagenesis, we discovered a novel gene, *GDDT*, which plays a dual role in plant biology: it acts as a positive regulator of growth and development, but as a negative regulator of drought resistance. The *gddt* mutant displayed a marked reduction in plant growth and seed setting rate, yet exhibited an unexpected advantage in terms of drought tolerance. Our research revealed that the enhanced drought tolerance of the *gddt* mutant is primarily due to a decrease in stomatal size, density, and aperture, which reduces water loss, and an activation of the reactive oxygen species (ROS) scavenging system, which helps protect the plant from oxidative stress. These physiological changes are observed both under drought conditions and in normal growth conditions. This discovery highlights the importance of *GDDT* as a pleiotropic gene with significant implications for both plant growth and drought resistance. Through map-based cloning, we determined that the protein disulfide isomerase-like (PDIL) gene *OsPDIL1-5* is the *GDDT* gene. The protein encoded by this gene was localized to the endoplasmic reticulum, consistent with its predicted function. Our findings provide new insights into the role of PDIL genes in rice and suggest that further study of *GDDT* could lead to a better understanding of how these genes contribute to the complex interplay between plant growth, development, and stress responses. This knowledge could pave the way for the development of rice varieties that are more resilient to drought, thereby increasing crop yields and ensuring food security in water-limited environments.

## Introduction

Water shortage is a global problem in agricultural production. In China, approximately 43% of the area is arid or semi-arid, and there is a great imbalance in the time-and-space distribution of water resources, leading to a contradiction between the water supply and demand in agricultural production. Rice is an important staple food crop. More than half of the world’s population rely on rice as the main food source (Tanaka et al., 2008). Using drought-tolerant rice varieties can greatly save water resources, stabilize food production, save energy, and reduce environmental pollution. Mining drought tolerance genes and understanding drought tolerance mechanism will facilitate the breeding of drought-tolerant varieties in rice.

Plants respond to drought stress through various physiological and biochemical regulatory mechanisms, such as specific signal transduction and osmotic pressure regulation. Adversity triggers the production of reactive oxygen species (ROS) molecules in plants, leading to their continuous accumulation beyond normal levels. The ROS accumulation causes membrane lipid peroxidation, generation of malondialdehyde (MDA), and irreversible damage to cell structures (Zorov et al., 2014). To counteract ROS accumulation, plants have developed a ROS scavenging system that includes antioxidant protective enzymes such as superoxide dismutase (SOD), peroxidase (POD), and catalase (CAT), as well as non-enzyme substances like reduced glutathione (GSH) and ascorbic acid (AsA). By eliminating ROS produced through various metabolic reactions, the ROS scavenging system maintains homeostasis to ensure the healthy growth and development of plants (Wang and Li, 2008). Reduced AsA can be produced from oxidized ascorbate (dehydroascorbate, DHA) through the catalytic action of dehydroascorbate reductase (DHAR), which relies on GSH. Therefore, DHAR plays a crucial role in the recycling of ascorbate for ROS detoxification (Do et al., 2016). In addition, stomata, which serve as the primary avenue for gas exchange between leaves and the external environment, play a crucial role in regulating photosynthesis, respiration, and transpiration and are closely associated with drought tolerance in plants (Huang et al., 2009). A lower stomatal density and reduced stomatal aperture can decrease transpiration rates, thereby enhancing drought tolerance.

Several genes that govern drought tolerance have been successfully cloned in rice. For example, *DST* controls stomatal closure and drought tolerance by regulating the accumulation of H_2_O_2_ (Huang et al., 2009). *DRO1* enhances drought tolerance by governing root growth angle and modifying root structure (Uga et al., 2013). *DSM1* modulates plant response to drought stress by scavenging ROS (Ning et al., 2010). *DS8* plays a crucial role in actin filament activity and influences drought sensitivity through ABA-mediated stomatal closure (Huang et al., 2019). *DHS* negatively regulates epidermal wax biosynthesis by promoting ROC4 degradation to enhance drought tolerance (Wang et al., 2018).

Plants often respond to environmental stress at the expense of growth and development, necessitating a delicate balance between growth and stress adaptation in adverse conditions. Many genes that govern plant growth and development also play a role in enhancing plant adaptability to the environment. These genes are vital for the survival of plant species and hold significant value for agricultural production. In rice, numerous genes controlling plant height have been identified, such as *D35*, *SD1*, *D18*, *D1*, *GID2*, *SLR1*, *BRD1*, and *D61* (Kurata et al., 2005). Mutations in these genes lead to reduced plant height, thereby improving resistance to lodging. Among them, the mutant allele *sd1* is a well-known Green Revolution gene that enables rice plants to withstand high fertilizer levels without lodging, ultimately boosting yields (Peng et al., 2021). Another beneficial gene in rice is *IPA1*/*OsSPL14*, which encodes a transcription factor with the SBP domain and is regulated by *OsmiR156*. Disrupting the inhibition of *OsmiR156* on *OsSPL14* through mutation results in a phenotype characterized by reduced tillering, lodging resistance, and high yield, known as the ideal plant type (Jiao et al., 2010).

Through irradiation mutagenesis, we obtained a single-gene recessive mutant of rice. This mutant displayed diminished plant growth yet increased drought tolerance, indicating that it is a crucial gene with pleiotropic effects on growth, development, and environmental adaptability. The objective of this study was to comprehensively investigate the mutant phenotype, identify the target gene via map-based cloning, and preliminarily elucidate the molecular and physiological mechanisms underpinning drought tolerance in the mutant. These findings establish a basis for future research on the role of the gene in growth, development, and drought tolerance in rice.

## Materials and methods

### Plant materials

The plant materials used in this study were: *indica* rice variety Huanghuazhan (HHZ); mutant *gddt* (*growth-depressed and drought-tolerant*) generated from HHZ through radiation mutagenesis; *japonica* rice variety Zhonghua 11 (ZH11); F_2_ populations derived from the crosses of *gddt* × ZH11 and *gddt* × HHZ, respectively.

### Investigation of agronomic traits

Seeds of HHZ (wild-type control) and *gddt* were sown individually in 96-well hydroponic boxes containing Kimora B formula nutrient solution, which was refreshed every 7 days. Shoot length and root length of seedlings were measured at the three-leaf stage. Additionally, HHZ and *gddt* were directly seeded in the paddy field at our university’s Jinshan campus in Fuzhou, following standard water, fertilizer, and pesticide management practices. At the mature stage, plant height, tiller number, panicle length, and seed setting rate were examined.

### Identification of seedling drought tolerance

Seeds of HHZ and *gddt* were planted in rectangular plastic trays (36 cm × 28 cm × 4.5 cm) filled with paddy soil. After reaching the three-leaf stage, seedlings were subjected to approximately 10 d of drought stress by withholding watering. Subsequently, seedlings were rehydrated (1.5 L/tray), and their survival rates were assessed.

### Microscopic observation of leaves

Seedlings of HHZ and *gddt* were cultured in 96-well hydroponic boxes as previously described. When reaching the three-leaf stage, seedlings were exposed to 20% PEG6000 for either 0 or 3 d. Subsequently, a 0.5 cm segment was cut from the middle part of the third leaf of each seedling, fixed with glutaraldehyde, washed with buffer solution, subjected to ethanol gradient dehydration and tert-butanol replacement, and finally dried using a VFD-21S freeze-drying instrument. Samples were then observed and photographed using a Leica S-800 scanning electron microscope. Stoma observation was conducted across 10 different visual fields for each sample.

### Measurement of physiological indexes related to drought tolerance

The hydrogen peroxide (H_2_O_2_) levels in the shoots of three-leaf seedlings of HHZ and *gddt* obtained through 96-well hydroponic box culture as previously described were quantified using a kit from Beijing Bioss Biotechnology Co., LTD after treatment with 20% PEG6000 for 0 or 3 d. Three biological replicates were analyzed, each comprising 5 seedlings. In addition, nitrogen blue tetrazole (NBT) staining analysis was conducted on the seedlings, utilizing the third leaf of each seedling. Five biological replicates were examined, with one seedling per replicate. Furthermore, MDA levels (assessed via the thiobarbituric acid method), DHAR activities (Beijing Solarbio Technology Co., LTD. Kit), SOD activities (NBT method), and CAT activities (UV absorption method) in the shoots of the seedlings under PEG treatment were monitored daily until the 5^th^ day. Once more, three biological replicates were established, each comprising 5 seedlings.

### Expression analysis of DHAR, SOD and CAT related genes

Seedlings of HHZ and *gddt* were cultured in 96-well hydroponic boxes according to the previously outlined method. Upon reaching the three-leaf stage, shoots were harvested from a portion of the seedlings, immediately frozen with liquid nitrogen, and stored in an ultra-low-temperature refrigerator. The remaining seedlings underwent treatment with 20% PEG6000 for 24 h before being similarly frozen with liquid nitrogen. RNA extraction was carried out using a plant RNA small amount extraction kit (Guangzhou Meiji Biotechnology Co., LTD.). Subsequent reverse transcription and real-time fluorescence quantitative PCR (RT-qPCR) were performed utilizing kits from Shanghai Yeasen Biotechnology Co., LTD. Each sample was subjected to three biological replicates, with the *Actin* gene serving as the internal reference. Primer sequences are detailed in **Supplementary Table S1**.

### Genetic analysis and gene mapping by single-pool BSA-seq

An F_2_ population consisting of approximately 1000 plants derived from the cross between *gddt* and ZH11 was cultivated in our university experimental field. Around 60 d post-sowing, a subset of the F_2_ population (comprising 245 plants) was examined to determine the segregation ratio between normal and mutant plants. Subsequently, 193 mutant plants were chosen from the F_2_ population to establish a mutant DNA pool (designated as MZ-pool). This DNA pool, along with DNA samples from the two parental lines, was dispatched to Xiamen Jointgene Biotechnologies Co., LTD. for deep sequencing using Illumina’s Hiseq 2000 platform. SNPs and short InDels between the two parents were identified from the sequencing data, with reference to the genome of the rice variety Nipponbare (version IRGSP-1.0). Leveraging SNP and short InDel markers, the genomic region harboring the target gene was identified using the block regression mapping (BRM) method (Huang et al., 2020).

### Fine mapping and identification of candidate gene

Based on the findings and data derived from BSA-seq, InDel markers were developed within the genomic region predicted to harbor the target gene (**Supplementary Table S1**). Employing these InDel markers, along with some available RM-series SSR markers (https://www.gramene.org/microsat/ssr.html), fine mapping of the target gene was conducted by analyzing the recombination events between each marker and the target gene in mutant plants selected from the aforementioned F_2_ population.

To pinpoint the candidate gene, an F_2_ population consisting of approximately 500 plants derived from the cross between *gddt* and HHZ was developed. From this population, 100 mutant plants were selected to form a pool (designated as MH-pool), which was then subjected to deep sequencing using the same methodology as described previously. Utilizing the sequencing data from this DNA pool, in conjunction with the single-pool BSA-seq data mentioned above, the polymorphic site displaying the most extreme allele frequency bias within the fine mapped interval was identified, and the gene containing this site was designated as the candidate gene.

### Gene knockout and genetic complementation test

To knockout candidate gene *OsPDIL1-5* (LOC_Os06g06790), two targets within the gene were designed for CRISPR-Cas9 (**Supplementary Table S1**; **Supplementary Figure S1A**) using the CRISPR RGEN software (http://www.rgenome.net/), followed by the construction of CRISPR-Cas9 vectors (**Supplementary Figure S1B**) and transfer of the vectors into ZH11. To perform genetic complementation test, the coding sequence of *OsPDIL1-5* was amplified from HHZ cDNA using Phanta Max Super-Fidelity DNA Polymerase (Nanjing Vazyme Biotechnology Co., LTD.) and inserted into the UBI-driven vector pRHVcGFP (**Supplementary Figure S1C**), which was subsequently introduced into the *gddt* mutant. The agronomic traits and drought tolerance of both *OsPDIL1-5* knockout (KO) and complementation (COM) lines were assessed using the methodologies outlined above.

### Analysis of OsPDIL1-5 expression and subcellular localization

The expression of *OsPDIL1-5* in different tissues of HHZ, including root, stem and leaf at the seedling stage, and spikelet (before flowering) and developing seed (7 d after flowering) during the heading period, was detected using RT-qPCR. The methods for RNA extraction and RT-qPCR were the same as described above (**Supplementary Table S1**). To examine whether OsPDIL1-5 is localized in endoplasmic reticulum (ER) as usually expected for a PDI-like protein, the pRHVcGFP-*OsPDIL1-5* vector, which carried the fused gene *OsPDIL1-5*-*GFP* (**Supplementary Figure S1C**), was co-transferred with ER-RFP marker vector (HDEL-AtWAK2-RFP) into rice protoplasts using the PEG-mediated method, using the empty pRHVcGFP vector as negative control. Fluorescence in the transformed protoplasts was observed, and images were captured using a confocal laser scanning microscope (TCS SP8, Leica) with excitation wavelengths of 488 nm for GFP and 561 nm for the ER-RFP marker.

## Results

### Morphological phenotype of *gddt*

**Figure 1:**
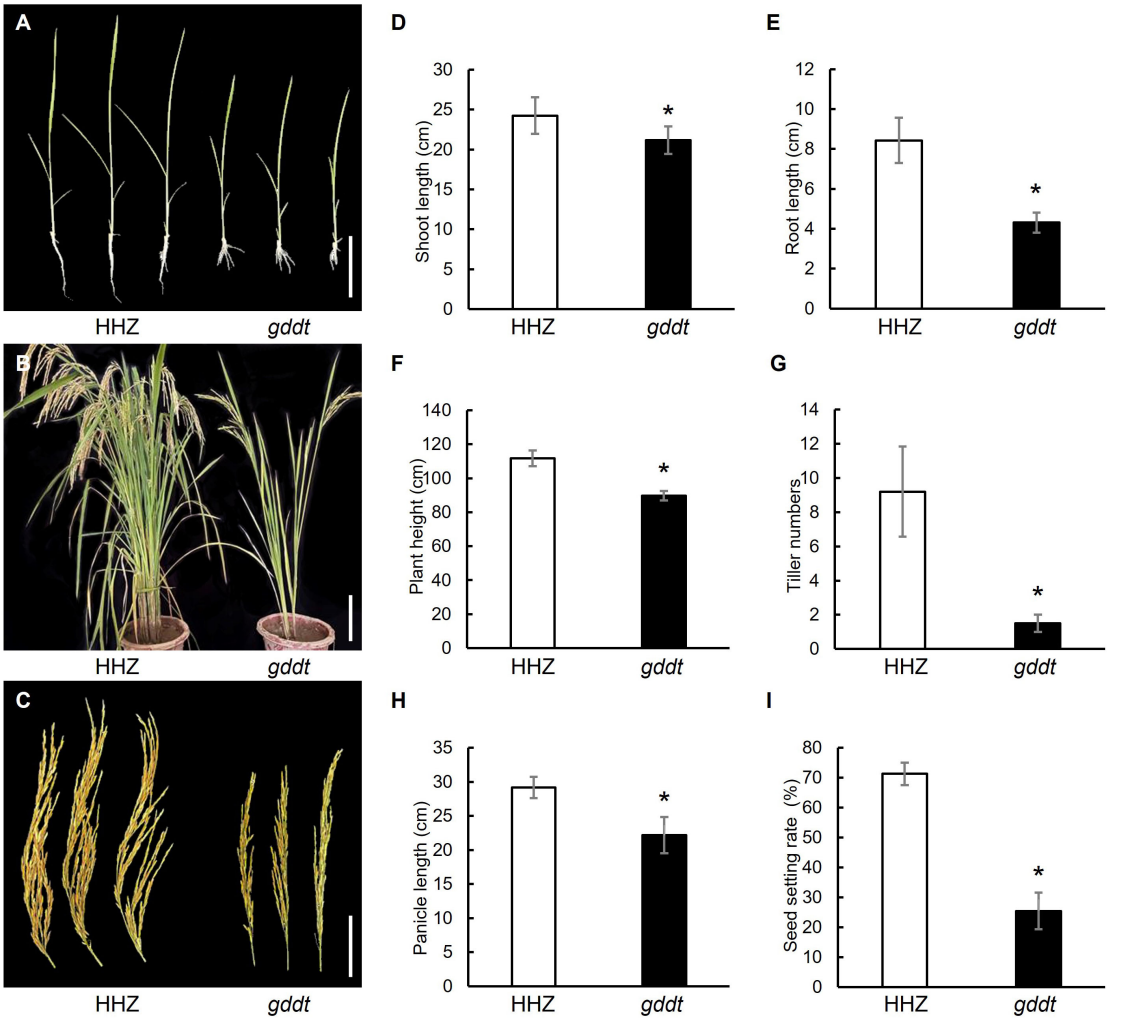
Comparison of agronomic traits in HHZ and *gddt.* **(A)** Two-leaf seedlings; Bar = 10 cm. **(B)** Mature plants; Bar = 30 cm. **(C)** Panicles; Bar = 10 cm. **(D–I)** Agronomic traits at seedling stage **(D, E)** or maturity stage **(F–I)**. The error bars indicate SD (n = 18); * indicates significant difference (*P* < 0.05) between HHZ and *gddt* detected by *t*-test.

Compared to HHZ, the *gddt* mutant exhibited a significant reduction in shoot length and root length at the seedling stage (**Figures 1A, D, E**) and in plant height, tiller number, panicle length, and seed setting rate at the maturity stage (**Figures 1B, C, F–I**), indicating that the mutation has a pleiotropic effect on both vegetative growth and reproductive development. Morphologically, the mutant could be easily identified by the naked eye based on the number of tillers (**Figure 1B**).

### Drought tolerance and leaf stomatal characteristics in *gddt* seedlings

**Figure 2:**
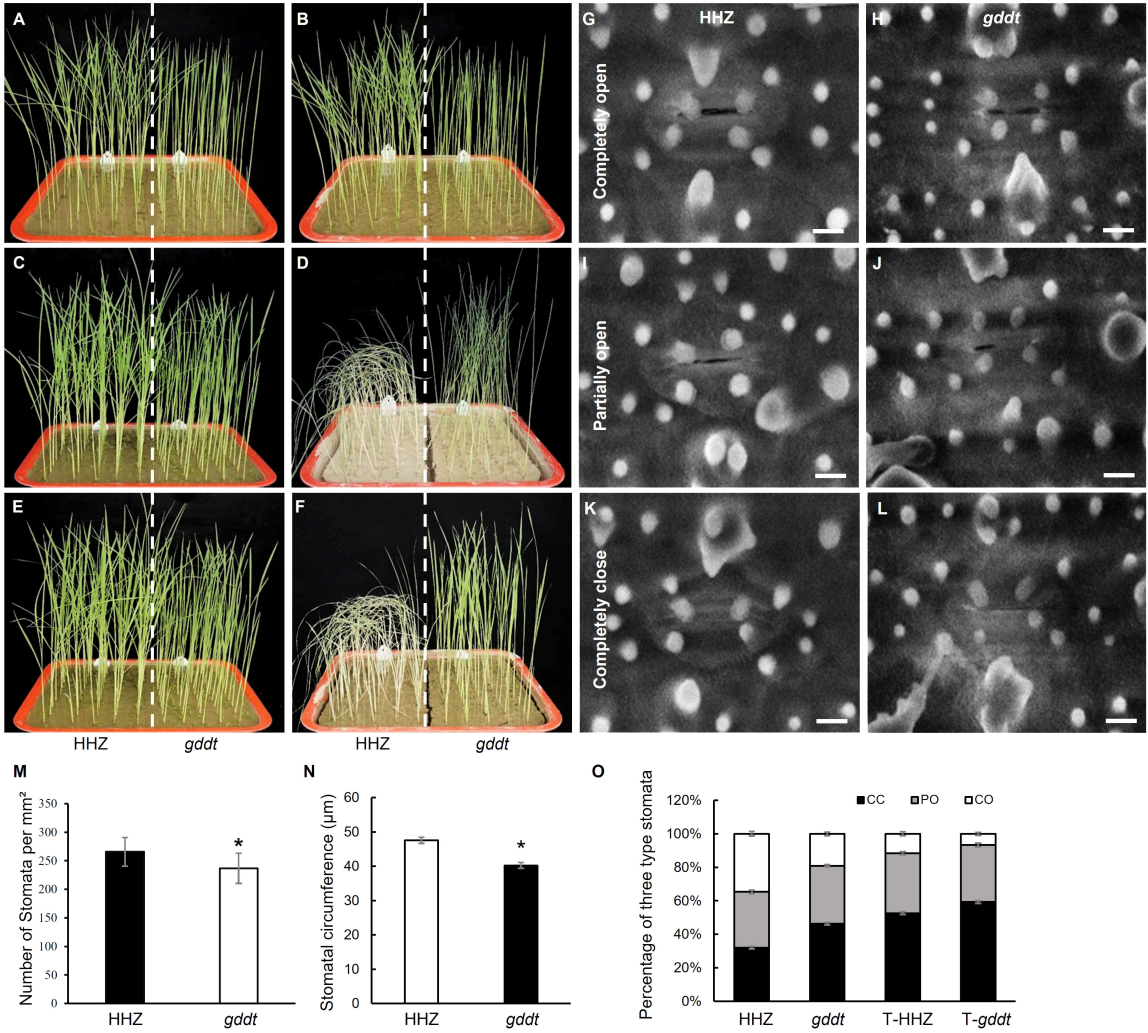
Drought tolerance and stomatal characteristics of *gddt* in comparison to HHZ at seedling stage. **(A, C, E)** Normal watering control group. **(B, D, F)** Drought stress treatment group. **(A, B)** Three-leaf seedlings before stopping watering. **(C, D)** 10 d after no watering. **(E, F)** 3 d after rewatering. **(G–L)** SEM images of stomata. Bar = 10 μm. **(M–O)** Stomata characters. T-HHZ and T-*gddt*, HHZ and *gddt* under drought treatment. CC, completely closed; PO, partially open; CO, completely open. The error bars indicate SD; n = 10 **(M, N)** or 9 **(O)**; * indicates significant difference (*P* < 0.05) between HHZ and *gddt* detected by *t*-test.

The performances of HHZ and *gddt* seedlings in the drought stress experiment are shown in **Figures 2A–F**. Following a period of no watering for 10 d, the HHZ seedlings wilted, while the *gddt* seedlings only displayed leaf curling (**Figure 2D**). After rewatering for 3 d, all the *gddt* seedlings recovered with leaves fully expanded, whereas only 3 (6%) HHZ seedlings recovered and survived (**Figure 2F**). These findings suggest that *gddt* is much more tolerant to drought than HHZ at the seedling stage.

Examination of abaxial leaf stomata using a scanning electron microscope revealed that *gddt* had smaller stomata than HHZ (**Figures 2G–L, N**). Additionally, *gddt* also exhibited a lower stomatal density than HHZ (**Figure 2M**). In regard to stomatal aperture, it could be classified into three categories in both *gddt* and HHZ: completely open (CO), partially open (PO), and completely closed (CC) (**Figures 2G–L**). Under normal conditions, *gddt* had a lower percentage of CO (denoted as CO%) but a higher CC% than HHZ, while the PO% was comparable between *gddt* and HHZ (**Figure 2O**). Under drought stress, the CO% decreased and the CC% increased in both *gddt* and HHZ compared to normal conditions, but the tendency of lower CO% and higher CC% in *gddt* than in HHZ was maintained, while the PO% was similar to that under normal conditions in both HHZ and *gddt* (**Figure 2O**). Overall, it can be inferred that *gddt* may primarily affect drought tolerance by causing a reduction in stomatal density, size, and aperture.

### Physiological indexes and gene expression related to drought tolerance in *gddt*

**Figure 3:**
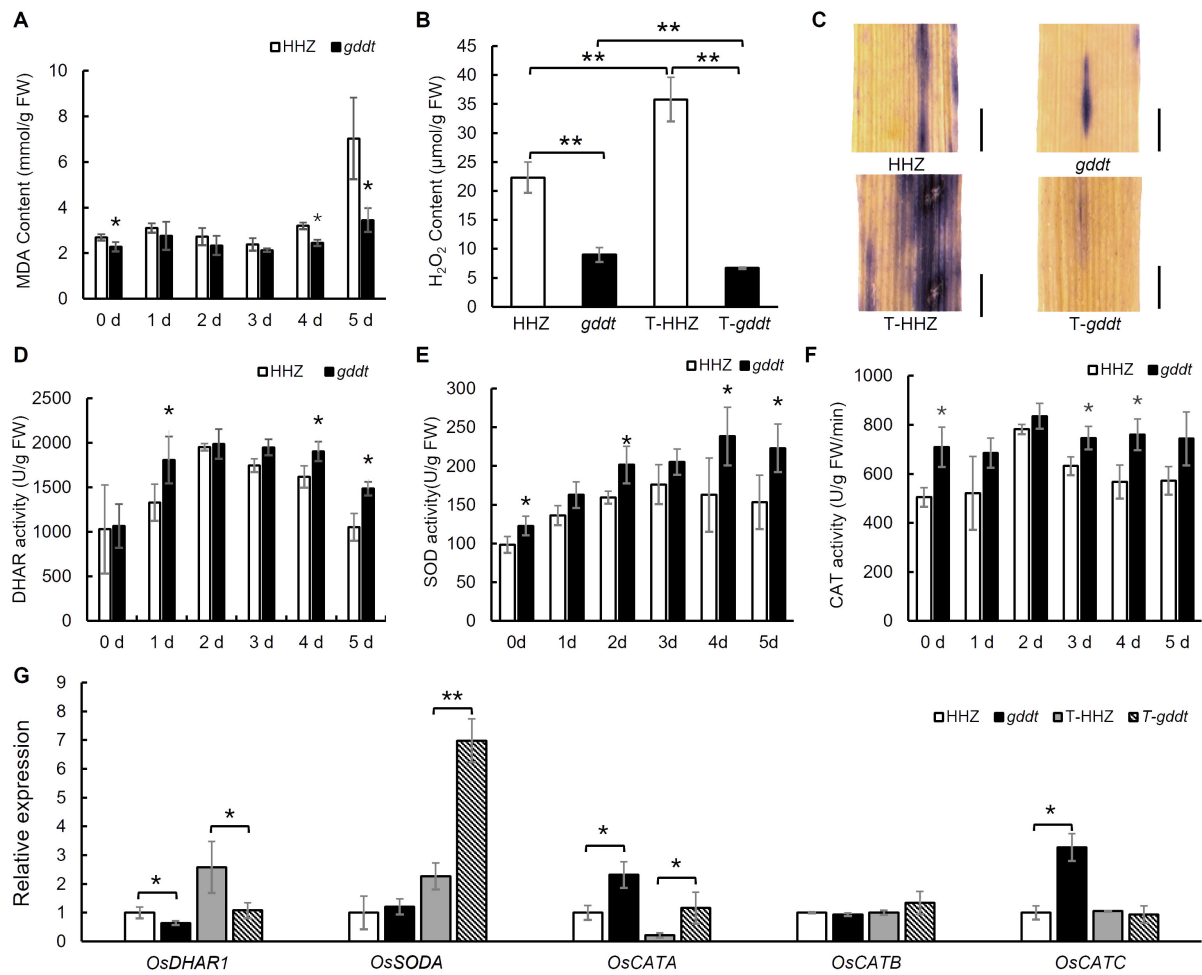
Physiological indices of drought tolerance and expression of related genes in *gddt*. **(A, D–F)** MDA content and DHAR, SOD and CAT activities under different days of drought stress. **(B, C)** H_2_O_2_ content and NBT staining (bar = 200 μm) before or after 3 d drought stress. **(G)** Relative expression of DHAR-, SOD-, and CAT-related genes before or after 24 h drought stress. T-HHZ and T-*gddt* indicate HHZ and *gddt* under drought treatment. The error bars in all the histograms indicate SD (n = 3); * and ** indicate significant difference detected by *t*-test at *P* < 0.05 and 0.01, respectively. In **(A, D–F)**, the *t*-test was performed between HHZ and *gddt* at each time point.

We analyzed five physiological indexes and the expression of five genes related to drought tolerance in both *gddt* and HHZ seedlings at the three-leaf stage. The MDA content was consistently lower in *gddt* compared to HHZ over the five-day stress period, with a notable difference on the 5^th^ day, aligning with the drought tolerance phenotype of *gddt*, although the difference was not statistically significant from the 1^st^ to the 3^rd^ day (**Figure 3A**). H_2_O_2_ content in *gddt* was significantly lower than in HHZ under both normal and drought conditions. Notably, after stress, H_2_O_2_ content increased in HHZ but decreased in *gddt* (**Figure 3B**), a trend confirmed by NBT staining (**Figure 3C**).

During the five-day drought stress period, DHAR activity initially increased and then decreased in both HHZ and *gddt*. However, the activity in *gddt* was consistently greater than or at least comparable to that in HHZ (**Figure 3D**). Similarly, SOD and CAT activities showed a pattern of initial increase followed by a decrease in both HHZ and *gddt* during drought stress, with *gddt* generally exhibiting higher values compared to HHZ (**Figures 3E, F**). RT-qPCR analysis indicated that the expression of SOD-encoding gene *OsSODA* increased in both HHZ and *gddt* after 24 h of drought stress, with a significantly higher relative expression level in *gddt* under drought conditions (**Figure 3G**), which aligns with the SOD activity observed. The relative expression levels of CAT isozyme genes *OsCATA* and *OsCATC* were higher in *gddt* than in HHZ under normal conditions, and *OsCATA* expression remained significantly higher in *gddt* than in HHZ after 24 h of drought stress (**Figure 3G**), paralleling the observed CAT activity. However, the expression pattern of the DHAR-related gene *OsDHAR1* did not well align with the DHAR activity. While the expression of *OsDHAR1* increased in both HHZ and *gddt* after 24 h of drought stress as expected, its relative expression level in *gddt* was lower than that in HHZ under both normal and drought conditions (**Figure 3G**). Nevertheless, despite this inconsistency, these findings suggest an overall activation of the ROS scavenging system in *gddt* to help mitigate drought stress.

### Inheritance of the mutant traits

Since tiller number is easily observed among all mutant traits, it was used as an index to analyze their heredity. The F_1_ generation of *gddt* × ZH11 displayed normal tillering, indicating that the mutant tiller phenotype (decreased tiller number) is recessive. Among the 245 F_2_ plants studied, 189 showed normal phenotype, while 56 displayed mutant phenotype, a ratio close to the theoretical 3:1 segregation (χ^2^ = 0.60, *P* = 0.439), suggesting that a single gene, named *GDDT*, controls the mutant traits.

### Location and candidate of *GDDT*

**Figure 4:**
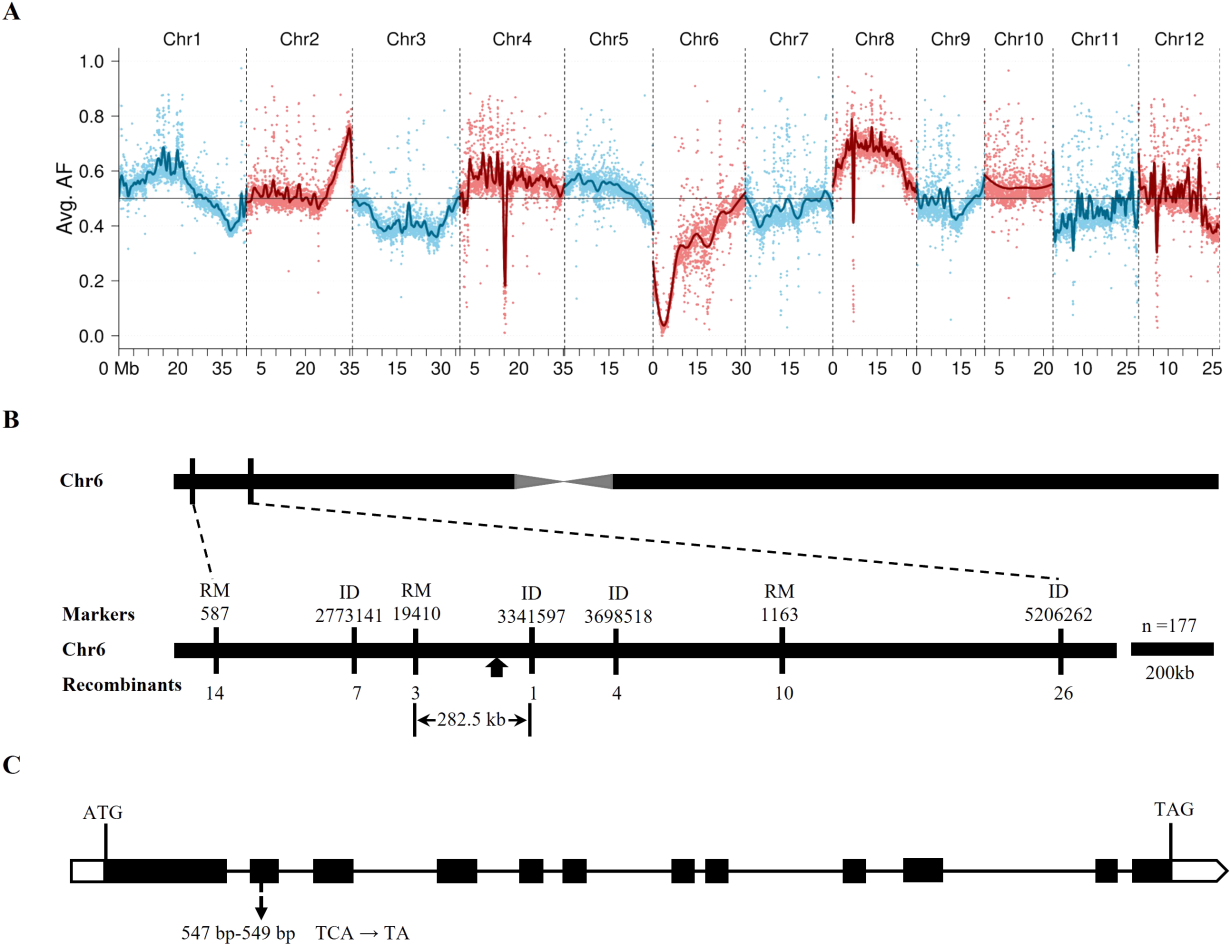
Mapping of *GDDT* and identification of its candidate gene. **(A)** The lowest wild-type allele frequency (AF) in the MZ-pool was detected on chromosome 6, indicating the region harboring *GDDT*. **(B)** Fine mapping delimited *GDDT* (indicated by filled arrow) between markers RM19410 and ID3341597. **(C)** Framework of *OsPDIL1-5* showing coding regions (filled boxes), UTRs (blank boxes), introns (horizontal lines), and the mutation site (indicated by arrow) with one base being deleted in the allele from *gddt* and displaying complete segregation distortion in both MH-pool and MZ-pool.

In the single-pool BSA-seq analysis, the mutant DNA pool (MZ-pool) and the parental DNA samples (*gddt* and ZH11) underwent deep sequencing, with average coverages of approximately 74.9, 137.2, and 141.2 times the rice genome, respectively. From the sequencing data, around 2.6 million SNPs and short InDels were identified between *gddt* and ZH11. BRM analysis using these markers revealed the lowest allele frequency in the MZ-pool around the 3 Mb position on chromosome 6 (**Figure 4A**), indicating a probable location for *GDDT*. Subsequent fine mapping based on 177 mutant plants from the F_2_ population of *gddt* × ZH11 using InDel markers and RM-series SSR markers narrowed down *GDDT* to an interval of 282.5 kb between markers RM19410 and ID3341597 (**Figure 4B**). Deep sequencing of the mutant DNA pool (MH-pool) from the F_2_ of *gddt* × HHZ revealed a single base deletion (TCA→TA) in the allele from *gddt* within the target interval at the position of 3,200,128 bp on chromosome 6. This mutation site displayed complete segregation distortion with only the deletion allele from *gddt* being detected in both MH-pool and MZ-pool, out of a total of 86 and 51 counts of the site, respectively. The deletion was located in the second exon of the *OsPDIL1-5* gene (**Figure 4C**), causing a frameshift mutation and early translation termination. Sequencing of *OsPDIL1-5* in *gddt*, HHZ, and three mutant plants randomly selected from the F_2_ population confirmed this deletion as the only mutation in the gene, implicating *OsPDIL1-5* as the most likely candidate gene for *GDDT*.

### Phenotype of *OsPDIL1-5* knockout lines

**Figure 5:**
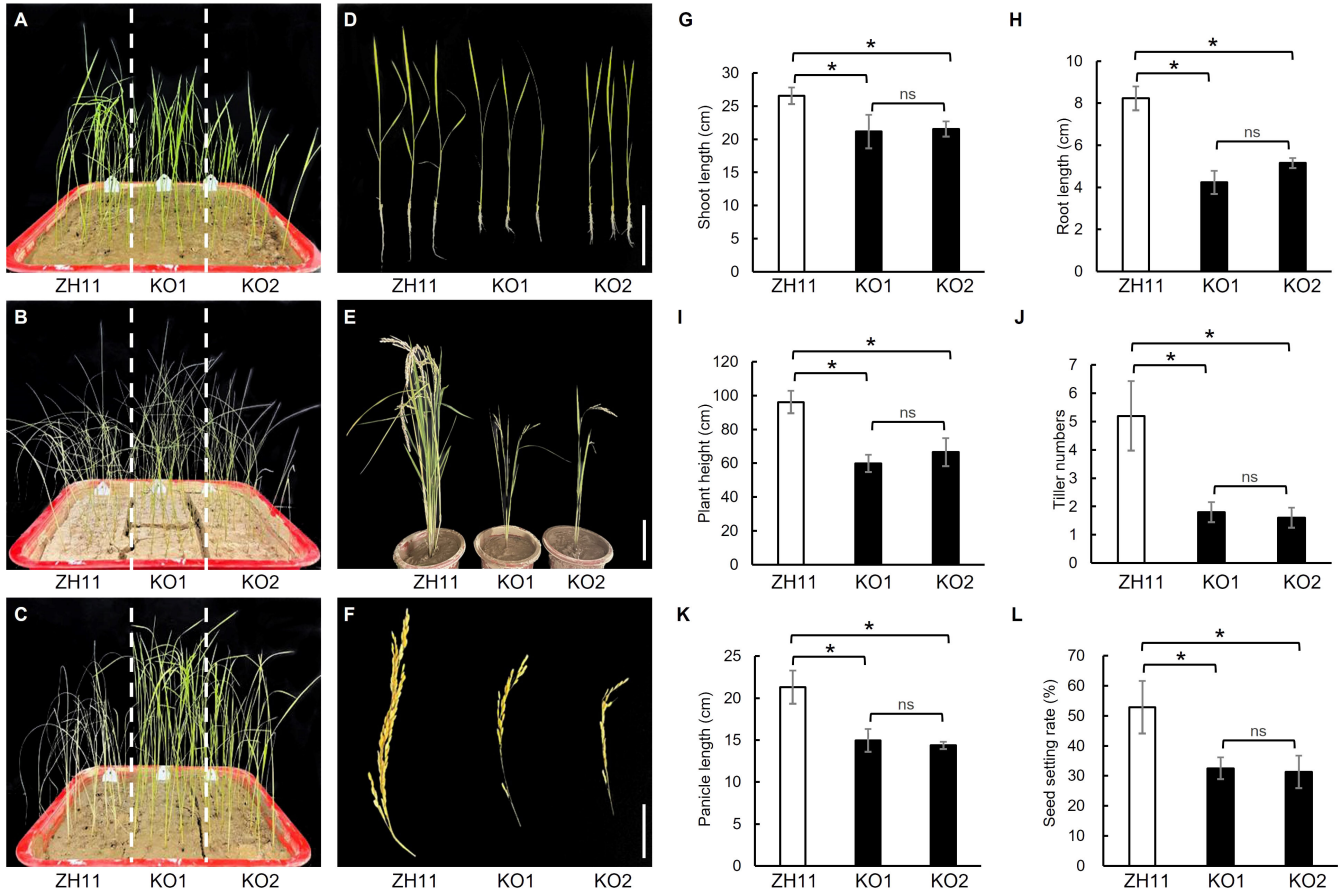
Comparison of drought tolerance and agronomic traits in ZH11 and two *OsPDIL1-5* KO lines. **(A–C)** Seedlings of before stopping watering **(A)**, 10 d after no watering **(B)**, and 3 d after rewatering **(C)**. **(D)** Two-leaf seedlings; Bar = 10 cm. **(E)** Mature plants; Bar = 30 cm. **(F)** Panicles; Bar = 10 cm. **(G–L)** Agronomic traits at the seedling stage **(G, H)** or maturity stage **(I–L)**. The error bars indicate SD (n = 18); * indicates significant difference at *P* < 0.05 detected by *t*-test; ns, not significant.

Two *OsPDIL1-5* knockout lines, *Ospdil1-5ko1* (KO1) and *Ospdil1-5ko2* (KO2), were generated from ZH11 using CRISPR-Cas9 (**Supplementary Figure S1A**). In KO1, a 209-base segment of *OsPDIL1-5* was deleted, starting from the 103^rd^ base in the first exon. In KO2, a one-base insertion occurred at the 312^th^ base in the first exon, leading to a frameshift mutation. Consequently, the function of *OsPDIL1-5* was completely impaired in both KO1 and KO2. In comparison to ZH11, KO1 and KO2 exhibited significantly enhanced drought tolerance and reduced shoot length and root length during the seedling stage (**Figures 5A–D, G, H**), as well as decreased plant height, tiller number, panicle length, and seed setting rate at the maturity stage (**Figures 5E, F, I–L**), mirroring the phenotypes of the *gddt* mutant. These findings provide confirmation that *OsPDIL1-5* is the target gene *GDDT*.

### Phenotype of *OsPDIL1-5* complementation lines

**Figure 6:**
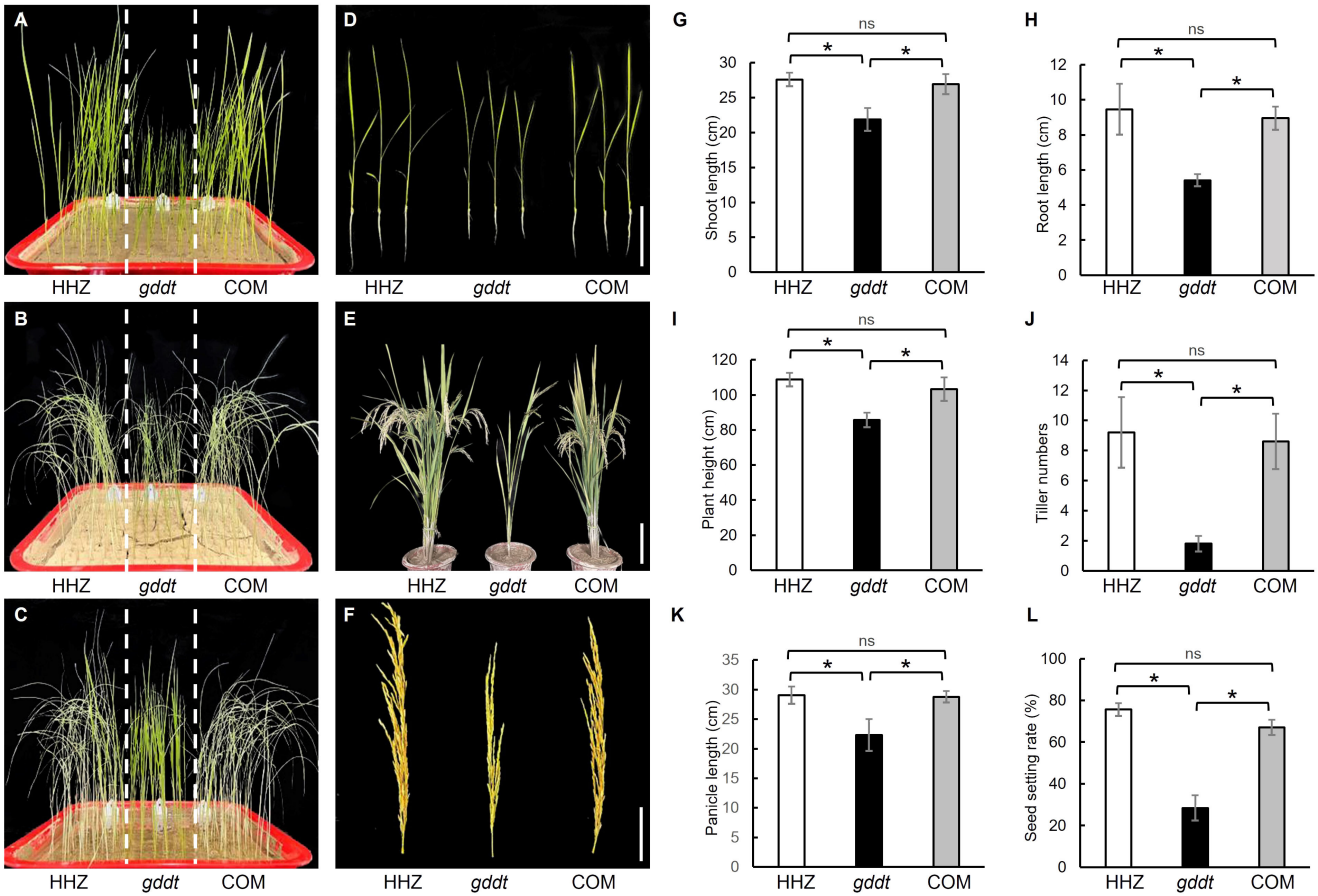
Comparison of drought tolerance and agronomic traits in HHZ, *gddt* and COM lines. **(A–C)** Seedlings of before stopping watering **(A)**, 10 d after no watering **(B)**, and 3 d after rewatering **(C)**. **(D)** Two-leaf seedlings; Bar = 10 cm. **(E)** Mature plants; Bar = 30 cm. **(F)** Panicles; Bar = 10 cm. **(G–L)** Agronomic traits at the seedling stage **(G, H)** or maturity stage **(I–L)**. The error bars indicate SD (n = 18); * indicates significant difference at *P* < 0.05 detected by *t*-test; ns, not significant.

Genetic complementation (COM) lines were generated by introducing the vector pRHVcGFP containing the coding sequence of the *OsPDIL1-5* gene into the *gddt* mutant (**Supplementary Figure S1**). Relative to *gddt*, the COM lines exhibited notably weaker drought tolerance (**Figures 6A–C**) and significantly longer shoot and root lengths (**Figures 6D, G, H**) during the seedling stage, as well as greater plant height, tiller number, panicle length, and seed setting rate at the maturity stage (**Figures 6E, F, I–L**), akin to the wild-type HHZ. This outcome provides further validation that *OsPDIL1-5* is indeed the target gene *GDDT*.

### Protein subcellular localization and expression pattern of *OsPDIL1-5*

**Figure 7:**
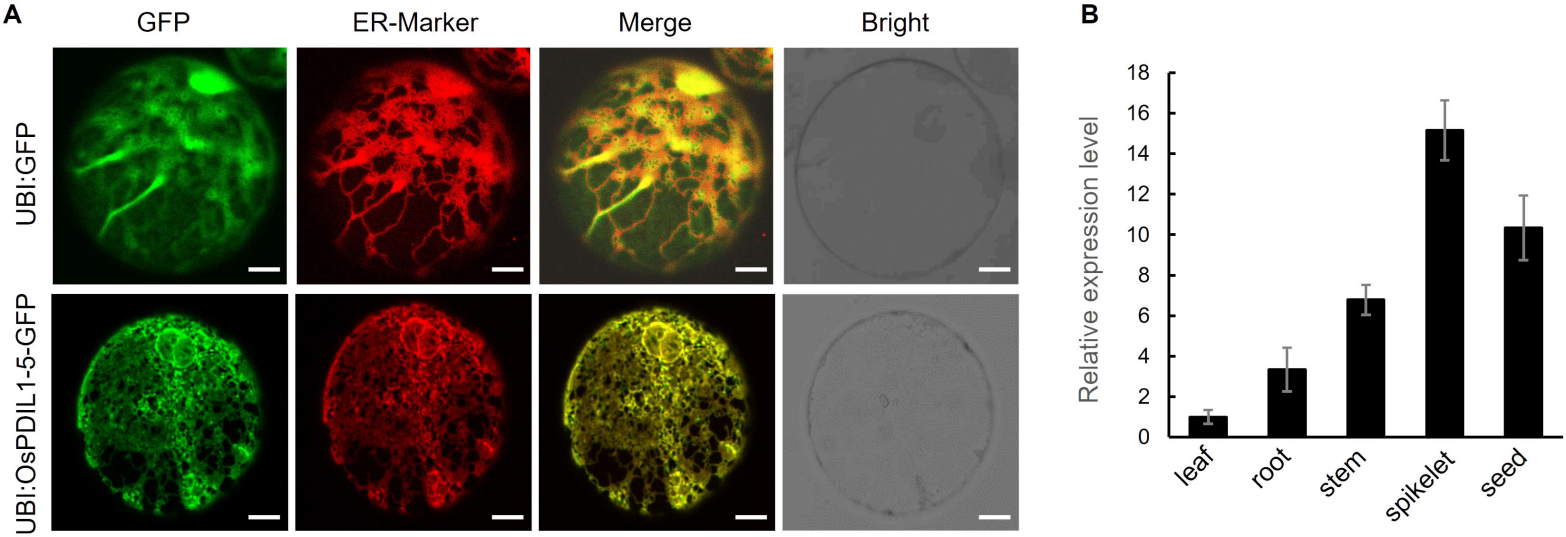
Subcellular localization of OsPDIL1-5 and expression pattern of *OsPDIL1-5*. **(A)** Subcellular localization of OsPDIL1-5. Bar = 7 μm. The marker (HDEL-AtWAK2-RFP) with red fluorescence localized in the ER. **(B)** Expression levels of *OsPDIL1-5* in different tissues. The error bars indicate SD (n = 3).

As expected, the merged images of green fluorescence from the OsPDIL1-5-GFP fusion protein and red fluorescence from the ER-marker observed under a confocal microscope clearly indicate that OsPDIL1-5 is specifically localized in the ER (**Figure 7A**).

RT-qPCR analysis revealed that *OsPDIL1-5* is expressed in all five tissues/organs assessed, with the highest expression in spikelet, followed by developing seed, stem, and root, and the lowest expression in leaf (**Figure 7B**).

## Discussion

In this study, we identified a novel gene of rice, *GDDT*, which is pivotal for the growth and development and drought tolerance, and determined it to be the *OsPDIL1-5* gene through map-based cloning. *OsPDIL1-5* is a member of the PDIL (PDI-like) gene family in rice (Houston et al., 2005). Protein disulfide isomerases (PDIs) are enzymes that assist in the oxidative folding of proteins within the ER by catalyzing the formation, reduction, and rearrangement of disulfide bonds (Urade, 2019). The molecular function of PDIs determines that the PDI gene family can influence various aspects of plant biology. The subcellular localization analysis confirmed that OsPDIL1-5 is localized within the ER (**Figure 7A**), which is consistent with its role as a PDI. The RT-qPCR analysis revealed that *OsPDIL1-5* is expressed in various tissues, including leaves, roots, stems, spikelets, and seeds (**Figure 7B**). This expression pattern implies involvement in both vegetative growth and reproductive development, supported by the mutant phenotype of *gddt*. In *gddt*, nearly all organs showed reduced size, and seed setting rate significantly decreased (**Figures 1**, **5**), highlighting *OsPDIL1-5* as a positive regulator of plant growth and spikelet fertility. Notably, *OsPDIL1-5* exhibited significantly varied expression levels among tissues, with the highest levels in spikelets and developing seeds, and lower levels in the vegetative tissues of seedlings (**Figure 7B**). This differential expression suggests a potentially more critical role for *OsPDIL1-5* in the reproductive phase compared to the vegetative phase.

The greater drought tolerance observed in the *gddt* mutant compared to the wild-type HHZ suggests that *OsPDIL1-5* acts as a negative regulator of drought tolerance. The microscopic observation of stomata and analysis of physiological indexes related to drought tolerance revealed that the *gddt* mutant likely enhances drought tolerance through two key mechanisms. Firstly, it reduces the density, size, and aperture of stomata (**Figure 2**), leading to a significant decrease in water evaporation from the plant through transpiration. Secondly, it activates the ROS scavenging system, effectively lowering intracellular ROS levels and protecting cells from oxidative damage (**Figure 3**). In addition, the reduced plant growth and biomass of the *gddt* mutant (**Figures 1**, **5**) may indirectly contribute to its improved drought tolerance by reducing water consumption for growth. Overall, the loss of *OsPDIL1-5* function inhibits plant growth, reduces stomatal density, size and aperture, boosts ROS scavenging system activity, and ultimately enhances drought tolerance.

Notably, while the wild type showed a marked increase in H_2_O_2_ content from normal to drought conditions, the *gddt* mutant exhibited a reverse trend, with H_2_O_2_ content decreasing further under drought conditions (**Figures 3B, C**). These results suggest that as a positive regulator of H_2_O_2_ accumulation, *OsPDIL1-5* has an even greater effect under drought stress. In addition, it is worth noting that, contrary to the typical response where elevated H_2_O_2_ levels lead to stomatal closure (Huang et al., 2009), *gddt* mutant plants had reduced H_2_O_2_ levels under both normal and drought conditions (**Figure 3B**), yet displayed higher rates of stomatal closure compared to wild-type plants (**Figure 2O**). This implies that *OsPDIL1-5* may also influence stomatal opening and closing through mechanisms unrelated to H_2_O_2_.

Environmental stress can disrupt protein folding, leading to ER stress, namely, an accumulation of misfolded or unfolded proteins in the ER. This triggers the unfolded protein response (UPR), a cellular mechanism aimed at restoring ER homeostasis (Carrillo et al., 2024). PDIs play critical roles in the UPR, functioning either as enzymes or as molecular chaperones essential for proper ER protein folding (Zhao et al., 2023). This underscores the significance of PDIs in plant tolerance to various environmental stresses, whether biotic or abiotic. Studies in *Arabidopsis* (Feldeverd et al., 2020; Zhang et al., 2018) and rice (Zhao et al., 2023) have demonstrated that loss of PDI gene function heightens sensitivity to environmental stress. However, an opposing finding has been observed in *Arabidopsis*, where the loss of *PDI5* gene function leads to increased proline accumulation at low water potential, thereby enhancing drought stress tolerance (Kumar et al., 2015). Similarly, in this study, the *OsPDIL1-5* gene exhibits a contrasting scenario: loss of *OsPDIL1-5* function improves, rather than diminishes, tolerance to drought stress. These findings suggest that while loss of PDI gene function is generally expected to increase susceptibility to stress due to impaired protein folding and compromised ER stress responses, it may, in some cases, paradoxically enhance stress tolerance through alternative mechanisms. In addition, although both *OsPDIL1-5* and *Arabidopsis PDI5* negatively regulate drought tolerance, they display opposite effects on plant growth, acting as a positive regulator and a negative regulator, respectively (Kumar et al., 2015). This further demonstrates the complexity of how PDI genes regulate plant growth and stress resistance.

The loss of PDI gene function has been shown to positively impact virus resistance as well. Studies indicate that suppressing PDI genes can hinder replication of human and animal viruses like dengue and influenza (Kim and Chang, 2018; Rawarak et al., 2019). Similarly, in plants, silencing *HvPDIL5-1* in barley (Yang et al., 2014) and *ZmPDIL-1* in maize (Dang et al., 2020) can increase resistance to Bymovirus and maize chlorotic mottle virus, respectively. However, contrasting scenarios also exist. For instance, silencing *SlPDI* in tomato plants heightens susceptibility to tomato yellow leaf curl virus, while its overexpression enhances virus resistance (Li et al., 2020). These findings underscore the diverse roles PDI genes play in plant virus resistance mechanisms.

In summary, the PDI gene family is vital for plant growth and responses to environmental stresses throughout all stages of plant development, highlighting its significant role. Different PDI genes have varying effects, either enhancing or diminishing traits, which underscores the complex mechanisms involved. The dual effects of a PDI gene on plant growth and stress resistance can complicate its use in breeding, especially when a gene affects these aspects in opposing ways, as observed with *OsPDIL1-5* in this study. Therefore, balancing the impact of the PDI gene on both growth and stress resistance is crucial. This balance might be achieved through genetic manipulation, such as modifying the gene’s expression and protein function using CRISPR/Cas9. We anticipate that rice varieties with moderately reduced *OsPDIL1-5* gene function may exhibit slightly lower yields under normal growth conditions, but could demonstrate significant yield advantages in arid environments, making them particularly suitable for cultivation in water-scarce regions. Currently, research on PDI genes in plants remains limited. The findings of this study provide valuable insights into the functions and mechanisms of PDI genes, laying a solid foundation for future in-depth research and potential modification of the *OsPDIL1-5* gene for rice breeding.

## Data Availability

The original contributions presented in the study are included in the article/**Supplementary Material**, further inquiries can be directed to the corresponding author/s.

## References

[B1] Carrillo R. Iwai K. Albertson A. Dang G. Christopher D. A. (2024). Protein disulfide isomerase-9 interacts with the lumenal region of the transmembrane endoplasmic reticulum stress sensor kinase, IRE1, to modulate the unfolded protein response in *Arabidopsis* . Front. Plant Sci. 15, 1389658. doi: 10.3389/fpls.2024.1389658 38817940 PMC11137178

[B2] Dang M. Q. Cheng Q. Hu Y. Wu J. X. Zhou X. P. Qian Y. J. (2020). Proteomic changes during MCMV infection revealed by iTRAQ quantitative proteomic analysis in maize. Int. J. Mol. Sci. 21, 35. doi: 10.3390/ijms21010035 PMC6981863 31861651

[B3] Do H. Kim I.-S. Jeon B. W. Lee C. W. Park A. K. Wi A. R.. (2016). Structural understanding of the recycling of oxidized ascorbate by dehydroascorbate reductase (OsDHAR) from *Oryza sativa* L. *japonica* . Sci. Rep. 6, 19498. doi: 10.1038/srep19498 26775680 PMC4726096

[B4] Feldeverd E. Porter B. W. Yuen C. Y. L. Iwai K. Carrillo R. Smith T.. (2020). The Arabidopsis protein disulfide isomerase subfamily M isoform, PDI9, localizes to the endoplasmic reticulum and influences pollen viability and proper formation of the pollen exine during heat stress. Front. Plant Sci. 11, 610052. doi: 10.3389/fpls.2020.610052 33447253 PMC7802077

[B5] Houston N. L. Fan C. Xiang J. Q. Schulze J. M. Jung R. Boston R. S. (2005). Phylogenetic analyses identify 10 classes of the protein disulfide isomerase family in plants, including single-domain protein disulfide isomerase-related proteins. Plant Physiol. 137, 762–778. doi: 10.1104/pp.104.056507 15684019 PMC1065376

[B6] Huang L. Chen L. Wang L. Yang Y. Rao Y. Ren D.. (2019). A Nck-associated protein 1-like protein affects drought sensitivity by its involvement in leaf epidermal development and stomatal closure in rice. Plant J. 98, 884–897. doi: 10.1111/tpj.2019.98.issue-5 30771248 PMC6849750

[B7] Huang L. Tang W. Bu S. Wu W. (2020). BRM: a statistical method for QTL mapping based on bulked segregant analysis by deep sequencing. Bioinformatics 36, 2150–2156. doi: 10.1093/bioinformatics/btz861 31742317

[B8] Huang X. Y. Chao D. Y. Gao J. P. Zhu M. Z. Shi M. Lin H. X. (2009). A previously unknown zinc finger protein, DST, regulates drought and salt tolerance in rice via stomatal aperture control. Gene Dev. 23, 1805–1817. doi: 10.1101/gad.1812409 19651988 PMC2720257

[B9] Jiao Y. Wang Y. Xue D. Wang J. Yan M. Liu G.. (2010). Regulation of OsSPL14 by OsmiR156 defines ideal plant architecture in rice. Nat. Genet. 42, 541–544. doi: 10.1038/ng.591 20495565

[B10] Kim Y. Chang K. O. (2018). Protein disulfide isomerases as potential therapeutic targets for influenza A and B viruses. Virus Res. 247, 26–33. doi: 10.1016/j.virusres.2018.01.010 29382552 PMC5831498

[B11] Kumar M. N. Hsieh Y.-F. Verslues P. E. (2015). At14a-Like1 participates in membrane-associated mechanisms promoting growth during drought in Arabidopsis thaliana. Proc. Natl. Acad. Sci. U.S.A. 112, 10545–10550. doi: 10.1073/pnas.1510140112 26240315 PMC4547278

[B12] Kurata N. Miyoshi K. Nonomura K. Yamazaki Y. Ito Y. (2005). Rice mutants and genes related to organ development, morphogenesis and physiological traits. Plant Cell Physiol. 46, 48–62. doi: 10.1093/pcp/pci506 15659430

[B13] Li T. Wang Y. H. Huang Y. Liu J. X. Xing G. M. Sun S.. (2020). A novel plant protein-disulfide isomerase participates in resistance response against the TYLCV in tomato. Planta 252, 25. doi: 10.1007/s00425-020-03430-1 32681182

[B14] Ning J. Li X. Hicks L. M. Xiong L. (2010). A Raf-like MAPKKK gene *DSM1* mediates drought resistance through reactive oxygen species scavenging in rice. Plant Physiol. 152, 876–890. doi: 10.1104/pp.109.149856 20007444 PMC2815886

[B15] Peng Y. L. Hu Y. G. Qian Q. Ren D. Y. (2021). Progress and prospect of breeding utilization of green revolution gene *SD1* in rice. Agriculture 11, 611. doi: 10.3390/agriculture11070611

[B16] Rawarak N. Suttitheptumrong A. Reamtong O. Boonnak K. Pattanakitsakul S. N. (2019). Protein disulfide isomerase inhibitor suppresses viral replication and production during antibody-dependent enhancement of dengue virus infection in human monocytic cells. Viruses 11, 155. doi: 10.3390/v11020155 30781856 PMC6410196

[B17] Tanaka T. Antonio B. A. Kikuchi S. Matsumoto T. Nagamura Y. Numa H.. (2008). The rice annotation project database (RAP-DB): 2008 update. Nucleic Acids Res. 36, D1028–D1033. doi: 10.1093/nar/gkm978 18089549 PMC2238920

[B18] Uga Y. Sugimoto K. Ogawa S. Rane J. Ishitani M. Hara N.. (2013). Control of root system architecture by DEEPER ROOTING 1 increases rice yield under drought conditions. Nat. Genet. 45, 1097–1102. doi: 10.1038/ng.2725 23913002

[B19] Urade R. (2019). Oxidative protein folding in the plant endoplasmic reticulum, Biosci. Biotechnol. Biochem. 83, 781–793. doi: 10.1080/09168451.2019.1571900 30712483

[B20] Wang Y. Li J. (2008). Molecular basis of plant architecture. Annu. Rev. Plant Biol. 59, 253–279. doi: 10.1146/annurev.arplant.59.032607.092902 18444901

[B21] Wang Z. Tian X. Zhao Q. Liu Z. Li X. Ren Y.. (2018). The E3 Ligase DROUGHT HYPERSENSITIVE negatively regulates cuticular wax biosynthesis by promoting the degradation of transcription factor ROC4 in Rice. Plant Cell 30, 228–244. doi: 10.1105/tpc.17.00823 29237723 PMC5810576

[B22] Yang P. Lüpken T. Habekuss A. Hensel G. Steuernagel B. Kilian B.. (2014). PROTEIN DISULFIDE ISOMERASE LIKE 5-1 is a susceptibility factor to plant viruses. Proc. Natl. Acad. Sci. U.S.A. 111, 2104–2109. doi: 10.1073/pnas.1320362111 24481254 PMC3926060

[B23] Zhang Z. R. Liu X. Li R. Yuan L. Dai Y. Q. Wang X. Y. (2018). Identification and functional analysis of a protein disulfide isomerase (*At*PDI1) in *Arabidopsis thaliana* . Front. Plant Sci. 9, 913. doi: 10.3389/fpls.2018.00913 30073003 PMC6060501

[B24] Zhao Q. Guan X. Zhou L. Xu Y. Asad M. A. U. Pan G.. (2023). *OsPDIL1-1* controls ROS generation by modulating NADPH oxidase in developing anthers to alter the susceptibility of floret fertility to heat for rice. Environ. Exp. Bot. 205, 105103. doi: 10.1016/j.envexpbot.2022.105103

[B25] Zorov D. B. Juhaszova M. Sollott S. J. (2014). Mitochondrial reactive oxygen species (ROS) and ROS-induced ROS release. Physiol. Rev. 94, 909–950. doi: 10.1152/physrev.00026.2013 24987008 PMC4101632

