# Supplementary material for: OsPDIL1-5: dual role in promoting growth and development while modulating drought stress tolerance in rice (Oryza sativa L.)
Source: Front Plant Sci. 2024 Oct 15;15:1479726. doi: 10.3389/fpls.2024.1479726 (PMC11518757; doi:10.3389/fpls.2024.1479726)
Supplement: Supplementary file 1 [file DataSheet1.docx]

**Supplementary Material**

**Table S1** Primers used in this study.

| Primer name | Primer sequences (5*'*~3*'*) | Application |  |
| --- | --- | --- | --- |
| *ACTIN* | TGGCATCTCTCAGCACATTCC  TGCACAATGGATGGGTCAGA | RT-qPCR |  |
| *OsDHAR1* | ATGGGCGTGGAGGTGTGCGTCAAGG  CCTTGCTCTTCAAGAACGTTGTGAAGC | RT-qPCR |  |
| *OsSODA* | ACAAATACCACCGCACACCG  GCACAACAACAGCCTTCACC | RT-qPCR |  |
| *OsCATA* | AGGAGGCAGAAGGCGACGATACA  TCTTCACATGCTTGGCTTCACGTT | RT-qPCR |  |
| *OsCATB* | GGCTGTCGGGAAAAGTGTGTCATTG  TTTCAGGTTGAGACGTGAAGCCAGC | RT-qPCR |  |
| *OsCATC* | TCAAGAGATGGATCGACGCACTCTC  GAAGCAGATTGCAACGCTGATCG | RT-qPCR |  |
| *OsPDIL1-5* | CCCAAGACAAAAGCAACCCG  ATGCCCGAAAGCTGTGAGAA | RT-qPCR |  |
| RM587 | TTCCCATCTGCACTACCATAATCC  GAGCAGAGATGTGCTTTGCTACC | SSR marker for locate (physical site 2291878) | |
| RM19410 | TGCTGATTGCTCACTACTTCATCC  GCGGGATACCATGGTCTAAAGG | SSR marker for locate (physical site 2913891) | |
| RM1163 | TGGACGCGGATAGGAGGAGACG  TCCTCCGCAAGGTCGGTTTCC | SSR marker for locate (physical site 4201371) | |
| Indel2773141 | CCACTGTACACAACTTGCATCC  TGAATGGTGTGGTGAAGGCT | Indel marker for locate (physical site 2773141) |  |
| Indel3341597 | AATCCTTGGAAAGGAGTGGCG  AACGTGTTTCAGAGAAGCATGG | Indel marker for locate (physical site 3341597) |  |
| Indel3698518 | AACAATCCGATTAGCAAGGACT  CGGCTTTCCTATTGACCCAGA | Indel marker for locate (physical site 3698518) |  |
| Indel5206262 | TTGCACTTAGGCCACTTTGCT  ACGCACAATCCGAGATCCAC | Indel marker for locate (physical site 5206262) |  |
| Target1 | CAGCGGGGACGACTCGGAGGTGC  AACGCACCTCCGAGTCGTCCCCG | Primer for target site 1 vector construction |  |
| Target2 | CAGGCAGCGCGCAGCTCATGCCG  AACCGGCATGAGCTGCGCGCTGC | Primer for target site 2 vector construction |  |
| *Hyg* | GAGCATATACGCCCGGAGTC  CAAGACCTGCCTGAAACCGA | Hygromycin screening transgenic plants |  |
| *KO*-seq | ATGAGGGCGCGGAGGGTTGT  GAGCAATTCCTGGGCCTTGT | The knockout experiment detects the mutation type |  |
| *OsPDIL1-5*-COM | CAGATCCAGTGGGATCATGAGGGCGCGGAG  ACTAGTGCGGCAAGCTTGGATGAGCTATAG | Clone *OsPDIL1-5* for complementary experiment |  |
| *OsPDIL1-5* | ATGAGGGCGCGGAGGGTTGTCG  CATTGTCAAGGATGAGCTATAG | Clone *OsPDIL1-5* CDS |  |

**
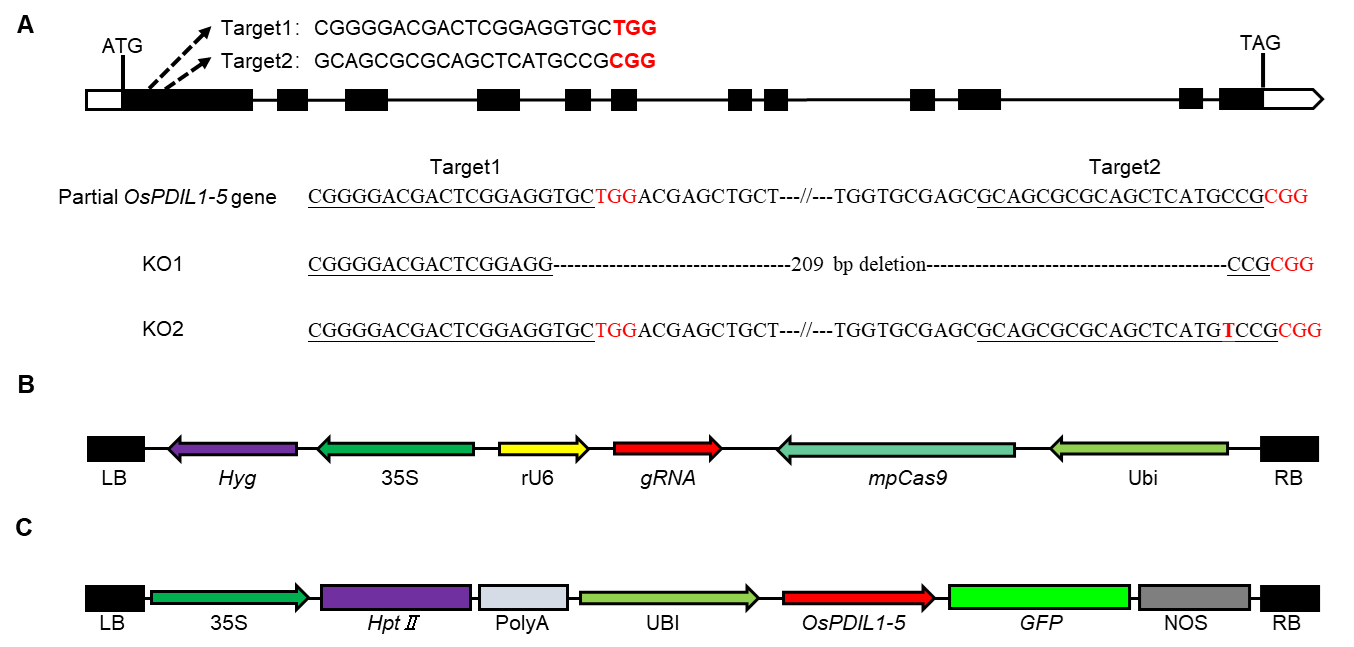
**

**Fig. S1.** Construction of vectors for *OsPDIL1-5* and knockouts obtained. (A) *OsPDIL1-5* gene framework, CRISPR/Cas9 target sites, and knockouts. Filled box, coding region; blank box, UTR; horizontal line, intron; underline, target site; red letters, PAM sites or inserted bases; KO1 and KO2, knockouts. (B) Diagram of CRISPR/Cas9 vector. (C) Diagram of overexpression vector pRHVcGFP-*OsPDIL1-5*.
